# The individual-cell-based cryo-chip for the cryopreservation, manipulation and observation of spatially identifiable cells. II: Functional activity of cryopreserved cells

**DOI:** 10.1186/1471-2121-11-83

**Published:** 2010-10-25

**Authors:** Elena Afrimzon, Naomi Zurgil, Yana Shafran, Friederike Ehrhart, Yaniv Namer, Sergei Moshkov, Maria Sobolev, Assaf Deutsch, Steffen Howitz, Martin Greuner, Michael Thaele, Ina Meiser, Heiko Zimmermann, Mordechai Deutsch

**Affiliations:** 1 Main Department for Biophysics and Cryotechnology, Fraunhofer IBMT, Ensheimer Straβe 48, 66386 St. Ingbert, Germany; 2 The Biophysical Interdisciplinary Schottenstein Center for the Research and Technology of the Cellome, Bar-Ilan University, Ramat Gan, 52900, Israel; 3 GeSiM mbH, Bautzner Landstraβe 45, 01454 Groβerkmannsdorf, Germany; 4 WBT Ltd., POB 1516, Ramat Gan, 52115, Israel; 5 Zentrum für gynäkologische Endokrinologie und Reproduktionsmedizin, Kaiserstrasse 5-7, 66111 Saarbrücken, Germany; 6 Professorship for Molecular and Cellular Biotechnology/Nanotechnology, University of Saarland, 66041 Saarbrücken, Germany

## Abstract

**Background:**

The cryopreservation and thawing processes are known to induce many deleterious effects in cells and might be detrimental to several cell types. There is an inherent variability in cellular responses among cell types and within individual cells of a given population with regard to their ability to endure the freezing and thawing process. The aim of this study was to evaluate the fate of cryopreserved cells within an optical cryo apparatus, the individual-cell-based cryo-chip (i3C), by monitoring several basic cellular functional activities at the resolution of individual cells.

**Results:**

In the present study, U937 cells underwent the freezing and thawing cycle in the i3C device. Then a panel of vital tests was performed, including the number of dead cells (PI staining), apoptotic rate (Annexin V staining), mitochondrial membrane potential (TMRM staining), cytoplasm membrane integrity and intracellular metabolism (FDA staining), as well as post-thawing cell proliferation assays. Cells that underwent the freezing - thawing cycle in i3C devices exhibited the same functional activity as control cells. Moreover, the combination of the multi-parametric analysis at a single cell resolution and the optical and biological features of the device enable an accurate determination of the functional status of individual cells and subsequent retrieval and utilization of the most valuable cells.

**Conclusions:**

The means and methodologies described here enable the freezing and thawing of spatially identifiable cells, as well as the efficient detection of viable, specific, highly biologically active cells for future applications.

## Background

The preservation of living cells has both theoretical and practical importance for human medicine, as well as for veterinary practice, cattle breeding, and plant cultivation [1-3]. The maintenance of intact living cells is necessary to preserve the cells' biological, biochemical, and functional properties as well as structural integrity, especially in rare and pedigree species. Of special interest is the preservation of definite sets of cells carrying specific antigens as well as stem cells. Such preservation opens wide possibilities for personalized therapy and for the development of innovative strategies for treating severe diseases, such as diabetes, nervous and cardio-vascular diseases, as well as various types of tumors [4-7].

The effects of freezing and thawing on the biophysical and biochemical features of living cells can result in alteration of their functional competency and survival [8]. Recently, numerous research groups have endeavored to optimize the procedure of freezing and thawing and to develop new cryopreservative media and instruments in order to minimize the damage caused by the freezing - thawing cycle (FTC) and to improve cell survival [1,9,10]. However, the ability to evaluate cell vitality potential, proliferation, and functional activity before and after cryopreservation is still limited.

We have recently introduced a novel device: the individual-cell-based cryo-chip (i3C) for the cryopreservation, manipulation and observation of spatially identifiable cells [11]. The aim of the present study is to evaluate the fate of cryopreserved cells in the device and to develop an approach for the efficient detection of viable, specific, highly biologically active cells for future applications. The results showed that U937 cells, after being thawed, have the same functional activities as non-frozen cells, confirming their biological integrity. Moreover, quantitative image analysis at the resolution of individual cells facilitated the detection of unique, bioactive single cells or cell groups, within heterogeneous cell populations.

## Methods

### The Individual-cell-based cryo-chip (i3C)

**Figure 1:**
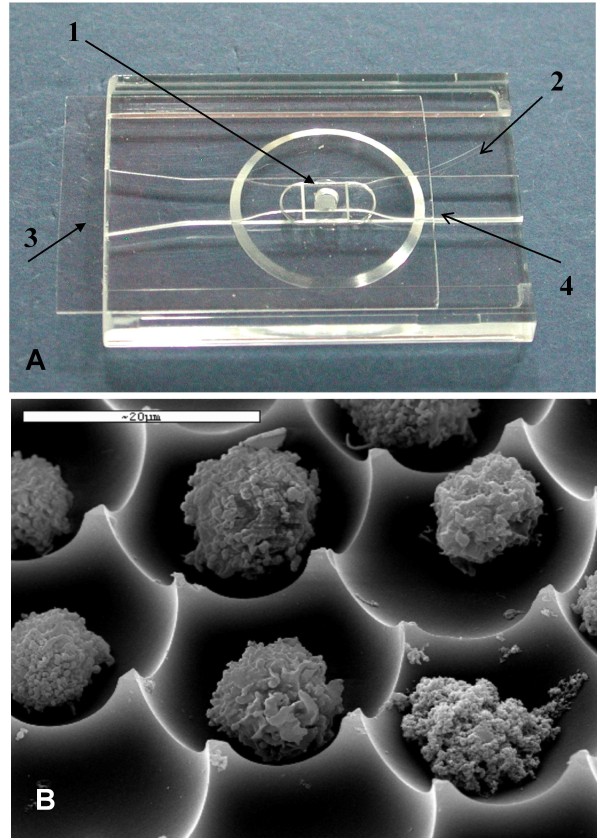
**The individual-cell-based cryo-chip (i3C)**. (A) The 2.5 ml cell chamber (1), whose glass bottom contains the picowells (small opaque region inside the chamber), which are engraved into the i3C plastic body (2). The cover slip (3) is moved left to enable access to the cell chamber opening, through which the cell suspension is loaded. The cover slip is then returned to its position in order to secure the content of the cell chamber when the liquids are poured into the entrance of the open conduit (4), formed by the space between the cover slip and the plastic 'step bridge'. Flow to the left is then established via capillary forces developed between the cover slip, the bridge and the poured liquid. (B) SEM micrograph of U937 cells in their picowells, as they appear at the bottom of the cell chamber.

The i3C is a configured micro-fluidics slide device, based on our previous work [12]. A detailed description of its mechanical and methodological aspects has been recently reported [11]. Briefly, the i3C is designed for holding individual cells or individual cell groups throughout cryopreservation, manipulation, and observation. The active area is a 2.5 μl chamber with a bottom which is made of densely packed transparent hexagonal matrix arrays of micron-sized wells (Figure 1). The high optical quality of the picowells enables high content image analysis of the cells maintained in the picowells, during their bio-manipulation, at an individual cell resolution.

### Biomaterials and probes

Propidium iodide (PI) and dimethyl sulphoxide (Me_2_SO) were obtained from Sigma-Aldrich (St.Louis, MO, USA). Annexin V - FITC Apoptosis detection kit was obtained from BioVision Inc. (Mountain View, CA, USA). Fluorescein diacetate (FDA) was purchased from Sigma-Riedel-de-Haen (Hanover, Germany). Tetramethylrhodamine methyl ester perchlorate (TMRM) was obtained from Molecular Probes (Eugene, Oregon, USA). RPMI-1640 medium, heat-inactivated fetal calf serum, penicillin, streptomycin, glutamine, sodium pyruvate and HEPES (all for the complete medium) were obtained from Biological Industries (Kibbutz Beit Haemek, Israel).

### Cells and culture media

Human pro-monocytic U937 cells (ECACC, UK) were maintained in RPMI-1640 medium supplemented with 10% heat-inactivated fetal calf serum (FCS), 100 U/ml penicillin, 100 μg/ml streptomycin, 2% glutamine, 2% sodium pyruvate and 2% HEPES. Cells were maintained in completely humidified air with 5% CO_2 _at 37°C. Before use, exponentially growing cells were obtained, washed, and resuspended in PBS at a concentration of 1.5-2.0 × 10^6 ^cells/ml. For post-thawing cell maintenance, the complete medium was supplemented by 20% FCS. The freezing - thawing cycle was performed in a cryo-protective medium supplemented by 20% FCS and 10% Me_2_SO.

### Experimental scheme

U937 cells at a concentration of 1.5-2.0 × 10^6 ^cells/ml underwent the freezing - thawing cycle in the i3C (experimental sample, 12 μl), in standard cryo-vials (control sample, 1 ml), or in mini-vials (control sample, 20 μl). The mini-vials were used as an additional control to test the effect of the cell suspension volume on the cryopreservation process. In order to avoid a potential diversity of results due to different cell concentrations, additional control samples were exposed to the freezing - thawing cycle in standard cryo-vials, using a regular cell concentration of 10 × 10^6 ^cells/ml.

In order to equalize the post-thawing experimental conditions, control samples (the cell suspensions that underwent the cryopreservation cycle within the cryo-vial), were loaded into the i3C after the freezing - thawing cycle. From that point onward, the cells that were cryopreserved in the i3C and in the cryo-vial experienced the same experimental and measurement conditions.

For cell division experiments, control samples (cells that underwent the cryopreservation cycle within standard cryo-vials) were transferred immediately after thawing to 10 ml fresh enriched medium in culture flasks. Untreated U937 cells were cultured in standard conditions as described above.

Two types of i3C were used in this study: 20 μm and 100 μm pitched. The former were used for single cell maintenance, while the latter were used for maintaining groups of cells in a single picowell.

### Cell loading

Cell loading into the i3C, whether for experimental samples or control samples, was carried out as follows: 5 μl of cell suspension (1.5 - 2 × 10^6 ^cells/ml) either in PBS or cryo-medium, was poured into the cell chamber and left for 7-10 min for cell sedimentation onto the picowell-padded bottom of the chamber. Next, 2.5 μl of supernatant medium was removed from the top of the suspension drop and the glass cover was closed, allowing for the introduction of a new medium portion (about 30 μl of total liquid volume) between the cover slip and the cell array conduit. The cell suspension in PBS was used for loading experimental samples into the i3C. After cell sedimentation, PBS was exchanged with the cryo-medium and the freezing - thawing cycle was started. For post-thawing loading of U937 cells from control cryo-devices, the cell suspension in the cryo-medium was loaded into the i3C as described above. Accordingly, the cryopreserved medium was exchanged with fresh complete medium.

### The Freezing - Thawing Cycle (FTC)

Experimental samples (i3C) and control cryo mini-vials underwent the freezing - thawing cycle either in a cryo-microscope or in a NALGENE Cryo 1°C Freezing Container (Thermo Fisher Scientific Inc., Waldham, MA, USA). An epifluorescence microscope (Eclipse 80i, Nikon, Japan) which was modified to include a cryostage (MDBCS 600, Linkam Instruments, UK) cooled with liquid nitrogen and heated with an electric heating wire was used. The cooling and heating rates were fully controlled by a TP94 controller. After 15 min of pre-cooling at 4°C, the freezing procedure was initiated and the samples underwent slow freezing at a rate of 1°C per minute. The freezing end point was -80°C. The control samples in standard cryo-vials were frozen in a NALGENE Cryo 1°C Freezing Container at the rate of about 1°C per minute until reaching -80°C in the Revco Refrigerator (Legaci Refrigeration System, Ashville, NC, USA). Samples were stored at-80°C for at least 15 min (in cryostage) and up to 4 days in the freezing container.

For long term storage under extremely low temperature conditions, the samples were transferred into liquid nitrogen after -80°C was reached. Samples were kept in liquid nitrogen (-196°C) for at least 2 days.

Thawing was performed as quickly as possible. Within the cryostage, the thawing of samples stored either in the i3C or in cryo mini-vials was always performed controllably: the heating rates were set between 40°C and 30°C per minute. The thawing of samples that were frozen in a standard NALGENE Cryo 1°C Freezing Container was conducted by placing the frozen sample in an incubator at 37°C for 3 minutes. On the average, the latter procedure quite reliably imitates the thawing conditions within the cryostage, as the temperature of the frozen sample is elevated from-80° to +37° in 3 minutes.

### Post-thawing culturing and staining

Following the freezing - thawing cycle, the cryo-medium was immediately exchanged with pre-warmed complete medium supplemented with 20% FCS. U937 cells were incubated in completely humidified air at 37°C with 5% CO_2 _for 1.5 hours, 3, 6, 24 and 48 hours. Cell staining was performed according to the protocols described below.

### Apoptosis screening after the freezing - thawing cycle

The early apoptotic events and cell viability were determined by double staining with an Annexin V and PI kit according to the manufacturer's protocol. U937 cells were washed with binding buffer and then FITC-Annexin V and PI were added as described in the instructions. Following 5 min incubation in the dark at RT, cells were evaluated by fluorescent microscope at two wavelengths.

### Cell proliferation after the freezing - thawing cycle

For cell proliferation experiments, the i3C with 100 μm picowells was chosen and U937 cells were loaded into the i3C before the freezing - thawing cycle. The cell proliferation was monitored for up to 48 hours, by tracing the number of cells within each individual group by transparent light imaging. The numbers of post-thawed U937 cells of the corresponding cell groups were counted immediately after thawing and after 48 hours.

The post-thawing cell proliferation in the i3C was compared both with the division of U937 cells underwent FTC in standard cryo-vials and then transferred to standard culture flasks as well as with the untreated U937 cells cultured under standard conditions as described above.

### Cytoplasm membrane integrity and metabolic activity of U937 cells after the freezing - thawing cycle

The rate of fluorescein-diacetate hydrolysis (FDA, 1.2 μM in PBS) by non-specific esterases was measured in U937 cells after thawing and was calculated from repeated periodical measurements (5 times, at time intervals of 60 sec) for the same cells [13]. A mixture of FDA (at a final concentration of 1.2 μM) and PI (at a final concentration of 2.5 μg/ml) was added to U937 cells loaded in the i3C at RT.

### Mitochondrial membrane potential screening in U937 cells after the freezing - thawing cycle

The mitochondrial membrane potential was assessed by TMRM staining [14]. Following the freezing - thawing cycle, U937 cells in the i3C were incubated in the presence of TMRM (final concentration of 200 nM, in PBS) for 15 min at 37°C, 5% CO_2_, and then washed with PBS.

### Multi-parametric analysis of cell physiological status after the freezing - thawing cycle

The simultaneous analysis of cells at different physiological statuses was performed using multi-parametric staining by TMRM, FDA, PI and Annexin V. Following the freezing - thawing cycle, U937 cells in the i3Cs were first stained by TMRM and by FITC-labeled Annexin V (as described above), and the fluorescent and transmitted light images were acquired. Then, a mixture of FDA and PI (see above) was introduced into the i3C and the same cells were imaged again. The kinetics of FDA hydrolysis was evaluated as described in the section *Cytoplasm membrane integrity and metabolic activity of U937 cells after the freezing - thawing cycle*. The coordinates for each acquired area (stage positions) were saved throughout the experiment. Thus, parallel analysis of mitochondrial membrane potential, intracellular esterase activity, and apoptosis levels were obtained for each individual cell.

### Measurements and image analysis

An Olympus Cell^R system mounted on an inverted IX81 microscope (Tokyo, Japan) was equipped with a sub-micron Marzhauser-Wetzlar motorized stage (type SCAN-IM, with an Lstep controller, Wetzlar-Steindorf, Germany), 14-bit ORCA II C4742-98 camera (Hamamatsu, Japan), and a filter wheel including fluorescence cubes (excitation filters, dichroic mirrors and emission filters, respectively: for fluorescein (i.e., FDA and Annexin V-FITC) 470-490 nm, 505 nm long pass, and 510-530 nm; for TMRM 510-560 nm, 565 nm, 577-632 nm; and for PI 540-580 nm, 595 nm, 600-660 nm. All filters were obtained from Chroma Technology Corporation (Brattleboro, VT, USA).

Olympus Cell^P software was used for image analysis. An in-house developed software package was used to monitor the picowells' structure for automatic cell recognition. This module automatically recognizes the picowells' structure, creates a region of interest (ROI) for each picowell and identifies the regions that are occupied by cells.

### Statistical analysis

For each experiment, 2-3 i3Cs were used. For each device, 4 images from 4 different areas were acquired (about 500-1000 individual cells total). For proliferation tests, at least 100 individual picowells/cell groups were analyzed. The mean and SD for each measured parameter were calculated for the different cell populations under investigation (controls and experiments). All the values are presented as average ± SD. The comparisons between experimental and control groups of cryo-devices were carried out using the ANOVA test for small sample groups.

## Results

### Cell viability after the freezing - thawing cycle

The most commonly used functional assay for cell viability is the assessment of plasma membrane integrity by PI exclusion test. Our preliminary results indicated that immediately after slow freezing and thawing, the viability of U937 cells (as measured by the percentage of PI negative cells) in the i3C was the same as in control samples, i.e., cells frozen in standard devices (the percentages of dead cells were 8.6% ± 3.5 and 9.1% ± 5.6, in the i3C and in standard vials, respectively). Since plasma membrane permeability is a late event in the process of cell death, and due to the fact that cell viability decreases during the time after thawing [15,16], cell death was examined after prolonged post-thawing incubation periods (Table 1).

**Table 1:** Cell death in U937 cells after the freezing - thawing cycle.

Time after thawing	The devices used for FTC			
	
	i3C	mini-vial	Standard cryo-vial	
			
			**2 × 10**^**6 **^**cells/ml**	**10 × 10**^**6**^**cells/ml**
1 hour (after-80°C)	8.7% ± 3.5		9.1% ± 5.6	

3 hours (after-80°C)	9.2% ± 5.1	6.3% ± 3.2	12.5% ± 4.1	

overnight (after-80°C)	23.4% ± 23.5	11.6% ± 2.3	19.6% ± 10.1	17.5% ± 11.3

overnight (after-196°C)	18.5 ± 2.9		24.6 ± 7.0	

U937 cells underwent a freezing - thawing cycle as described in *Materials and Methods. *The percentage of dead (PI positive) cells was tested at appropriate time points after thawing. The corresponding temperature end points are presented. Values are average ± SD of 2-3 experiments (500-1000 individual cells).

There was no increase observed in the number of dead cells during the first 3 hours after the freezing - thawing cycle, and it remained the same in experimental (i3C) and in cells frozen under standard conditions. Prolonged culturing of post-thawed U937 cells in fresh enriched medium overnight demonstrated an increase in the proportion of dead cells both in the experimental (i3C) and standard devices samples (Table 1). The percentage of dead cells (PI positive) post thawing in the i3C varied from 5% to 54% in different samples. The corresponding parameters in the standard device samples varied from 7% to 32% in the cryo-vial and from 7% to 13% in the mini-vial. The statistical differences were not significant (*P *= 0.5). The results of cell viability overnight after thawing were supported by the evaluation of the vitality of U937 cells which were frozen first at -80°C and then transferred to liquid nitrogen (-196°C). The cells frozen in the i3C demonstrated a better cell survival overnight after thawing in comparison to cells frozen in standard cryo-vials (*P *= 0.02). It should be noted that the level of cell death measured in untreated U937 cells which were cultured for 24 hours in i3Cs did not differ from that of untreated cells that were cultured under the same conditions in standard flasks (9.3% ± 9.2 and 4.7% ± 2.8 respectively, *P *> 0.2).

The post-thawing cell death in the current experimental procedures (cell concentration of 1.5 - 2 × 10^6^cell/ml, see *Cell loading *above) was compared to the death of cells frozen at a standard concentration in cryo-vials (10 × 10^6 ^cell/ml). No statistical difference was found overnight after thawing in the percentage of cell death between the two cryo-protocols, for the low and high cell concentrations, respectively (*P *= 0.7).

### Cell apoptosis after the freezing - thawing cycle

It has been shown that the low survival of human embryonic stem cells in conventional slow-cooling cryopreservation protocols is predominantly due to apoptosis rather than cellular necrosis [16]. Other cell types, such as bone marrow cells, also display a high level of apoptosis upon freezing and thawing [17].

The level of apoptosis in U937 cell populations which underwent freezing and thawing in the i3C or in control cryo-vials was determined by Annexin V binding to externalized phosphatidylserine. Under the current experimental conditions, variable percentages of early apoptotic cells (Annexin V positive and PI negative) within different cultures of the U937 pro-monocyte cell line were found both in untreated and in cryopreserved cells (Table 2). The average percentage of early apoptotic cells 3 hours after the freezing - thawing cycle was the same for cells in the experimental i3C and for control device samples (4.3% ± 2.1 and 4.6% ± 1.4, respectively, *P = *0.1). Wide-ranging results are shown following overnight incubation of either untreated or post-thawing U937 cells. However, the average proportion of early apoptotic cells did not differ significantly in untreated cells, cells that underwent FTC in the i3C, or in standard cryo-vials *(P *= 0. 2).

**Table 2:** Early apoptosis events in U937 cells after the freezing - thawing cycle.

Time after thawing	The devices used for FTC		Untreated cells*
		
	i3C	standard cryo-vial	
**3 hours**	4.3% ± 2.1 (3% - 7%)		9.9% ± 6.2 (1% - 19%)

**overnight**	10.0% ± 10.0 (2% - 20%)	3.0% ± 2.8 (1% - 5%)	7.0% ± 4.3 (1.5% - 10%)

U937 cells underwent a freezing - thawing cycle as described in *Materials and Methods. *The percentage of early apoptotic (Annexin V positive and PI negative) cells was tested at appropriate time points after thawing. Values are average ± SD of 2-3 experiments (500-1000 individual cells). The range of results obtained for the different experiments (as percentages) is presented in brackets. *Untreated U937 cells, which did not undergo FTC, were evaluated at the corresponding time points.

### Cell proliferation after the freezing - thawing cycle

Cell maintenance and growth depend on the ability of cells to divide and proliferate. Hence, apart from the cell intactness, the ability of cryopreserved cells to grow and proliferate is a major, essential factor for a successful cryopreservation process.

**Figure 2:**
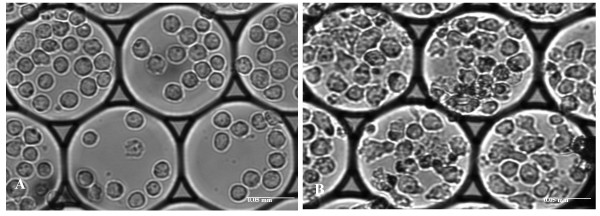
**Cell proliferation after thawing**. Bright field images of the cells (objective × 20) in the same individual picowells: A - immediately after the freezing - thawing cycle, B - following 48 hour culturing. Scale bar: 0.05 mm.

Cell division was evaluated using i3C devices with 100 μm picowells. The 100 μm diameter picowells in the i3C array are large enough to hold several individual cells (up to 20 cells) in each picowell. The direct counting of the number of individual cells within each group/picowell immediately and several times after cell thawing facilitates the estimation of the division capability of the post-thawed cells. Figure 2 shows transmitted light images of the same U937 cell groups, immediately after thawing and 48 hours after culturing. As can be seen, dramatic morphological changes are manifest upon cell growth and there is an increase in cell numbers.

Analyzing the same picowells immediately after thawing and 48 hours after thawing showed an increase in cell number from 9.6 ± 5.3 cells per picowell (total 1,061 individual cells) to 10.7 ± 4.6 cells per picowell (total 1,192 individual cells), yielding an average ratio of cell numbers 48 hours post-thawing to time 0 of 1.26 ± 0.71 as compared to a ratio of 1.53 ± 0.57 for U937 cells which underwent the freezing - thawing cycle at the same cell concentration within standard cryo-vials and were then transferred to culture flasks (no statistically significant differences, *P *= 0.1). It should be noted that the proportion of dead cells 48 hours after thawing was the same for cells frozen in standard cryo-vials and cultured for 48 hours in a flask and for experimental cells in the i3C (24.5% ± 3.44 and 23.45% ± 12.4, respectively, *P *= 0.7). Under standard culturing conditions, untreated U937 cells (1.5 × 10^6^/ml) double their number within 48 hours.

Analyzing cell division at the resolution of single picowells enabled us to select the specific cell groups that showed the highest proliferation rates. Indeed, in half of the picowells an increase in cell number was evident upon 48 hours of post thawing culturing (the ratio of cell number at 48 hours to time 0 was 1.58 ± 0.83), while the other cell subsets exhibited no change or a decrease in cell number. Such variation in cell division may be attributed to the different responses of non-synchronized cells to FTC, as previously described [18].

### Mitochondrial membrane potential (MMP) after the freezing - thawing cycle

Mitochondria are essential cell organelles involved in a variety of cellular activities, including ATP synthesis, intracellular calcium homeostasis, and both integration and execution of apoptotic signals. The mitochondrial membrane potential (MMP) is a major indicator of mitochondrial function. Higher MMP normally implies a better mitochondrial function [19]. Moreover, differences in MMP detected within and between cultured cells reflect the corresponding differences in mitochondrial function or levels of their activity [20].

**Figure 3:**
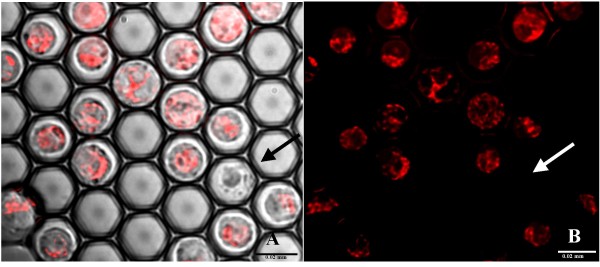
**Mitochondrial staining of untreated U937 cells by TMRM (200 nM), a potentiometric membrane dye which accumulates in the mitochondria**. Images (× 60 magnification): the overlay of transmitted light and fluorescent images (A) and the corresponding fluorescent image (B) are presented. The white arrow indicates a TMRM negative U937 cell, also characterized by its morphological appearance in the transmitted light image. Scale bar: 0.02 mm

In the present study, the mitochondrial membrane potential was evaluated by staining the cells with TMRM, a potentiometric membrane dye which accumulates in the mitochondria. In normally functioning TMRM stained cells, the intensity of the fluorescent signal is high and it is localized mainly within the mitochondria and not in the cytoplasm (Figure 3A,B). Hence, the mean fluorescence intensity of the TMRM signal over the cell area (mean FI) reflects the mitochondrial trans-membrane potential, while the dispersion of the TMRM signal within the cell region (FI Standard Deviation, FI SD) indicates the spatial distribution of TMRM staining. High FI SD designates heterogeneous staining, e.g., considerable differences between mitochondrial and cytoplasmic fluorescence intensity. Untreated, normal TMRM stained U937 cells are characterized by high values of both mean FI and FI SD reflecting their normal mitochondrial activity (average FI values 247.7 au ± 55.4 and average FI SD 172.2 ± 41.5).

The MMP after the freezing - thawing cycle was measured immediately after thawing (within 30 minutes), after 1.5, 3 and 6 hours.

All post-thawed U937 cells were TMRM positive within 30 min after thawing. However, the average FI of TMRM-stained cells immediately after thawing was low compared to unfrozen samples (*P *< 0.05), indicating a relatively low mitochondrial trans-membrane potential, both in the experimental i3C samples (average mean FI of 100.5 au ± 38.9 and average FI SD 150 au ± 99) and in control cells frozen in standard cryo-vials (average mean FI of 131.1 au ± 67.8 and average FI SD of 118.3 au ± 49.3). No statistically significant differences with regard to both parameters of TMRM staining were demonstrated between the i3C and samples that underwent FTC in a standard cryo-vial (*P *= 0.6).

**Figure 4:**
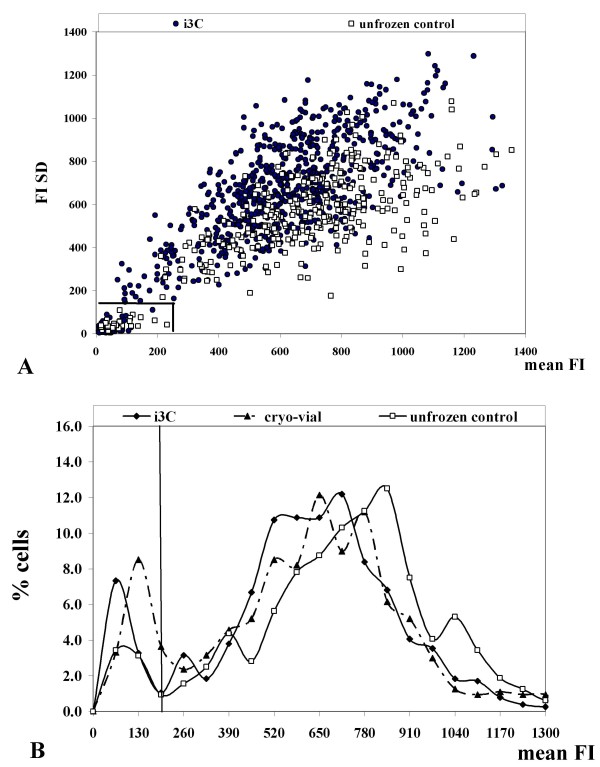
**Characteristics of the mitochondria in U937 cells after the freezing - thawing cycle (1.5 hours)**. (A). Scattered diagram of single cells' mean FI (X axis) vs. FI SD (Y axis) for individual cells stained with TMRM in the i3C (1.5 hours after the freezing - thawing cycle) and unfrozen control. Each dot represents an individual cell. (B) Distribution histograms of FI values in individual U937 cells that underwent the freezing - thawing cycle in the i3C, standard cryo-vials and the unfrozen control.

After 1.5 hours post-thawing, the U937 cells exhibited MMP values similar to those of control unfrozen cells. Figure 4A depicts a scatter diagram of mean FI vs. FI SD of individual U937 cells without freezing and 1.5 hours post-thawing incubation. It can be seen that both parameters are similar in unfrozen controls and in the experimental i3C cell populations. A small (4.2% ± 0.3) subset of TMRM negative cells (no detectable MMP) appeared among U937 cells after the 1.5 hour post-thawing period. The proportion of this TMRM negative cell group was 2.7% ± 1.9 and 6.3% ± 1.5 at 3 and 6 hours post-thawing, respectively. No statistically significant difference was demonstrated in the percentage of these TMRM-negative cells between the cryo-devices (*P *= 0.1-0.7). These results are in agreement with the above results that showed a decrease in cell viability during post-thawing incubation, as expressed by the PI exclusion test.

Distribution histograms of individual TMRM mean FI values within U937 cell populations exposed or unexposed to the freezing - thawing cycle are shown in Figure 4B. A wide distribution of individual cells' mitochondrial potential values is shown within the unexposed U937 cell population. Similar distributions of individual cells' FI values are evident after 1.5 hour incubation in the post-thawed cell groups (i3C and cryo-vials) indicating normal mitochondrial activity. Within the three cell populations, the cell subgroup which exhibited a low MMP (TMRM FI < 200 au) is evident. The percentage of these cells with a low mitochondrial activity increased following the freezing - thawing cycle in both cryopreservation protocols (12% and 16% in the i3C and cryo-vials, respectively), in respect to the untreated cell population (7%).

At this time (1.5 hours post-thawing), the mean FI and FI SD in the cell populations exposed to the freezing - thawing cycle either in the i3C or in the control cryo-vials (both mini-vials and standard vials) exhibited no statistically significant differences (Table 3). 24 hours after thawing, U937 cells in the i3C demonstrated full mitochondrial recovery and showed mitochondrial activity equal to that of the untreated cells, with an average mean FI of 527.7 au ± 506.6 and FI SD of 328.3 au ± 257.2 for the cells frozen in the i3C, and a mean FI of 346.6 au ± 345.6 and FI SD of 215.8 au ± 171.8 for the unfrozen control cells (*P *= 0.5 and 0.4, correspondingly).

**Table 3:** Mitochondrial membrane potential of U937 cells after the freezing - thawing cycle.

TMRM fluorescence signal	The devices used for FTC			*P*
		
	i3C	mini-vial	standard cryo-vial	
**mean FI (au)**	726 ± 578.8	754.5 ± 690.8	814.8 ± 534.4	0.9

**FI SD (au)**	532.8 ± 327.5	455 ± 332.3	624.5 ± 365.6	0.8

U937 cells underwent a freezing - thawing cycle in appropriate cryo-devices and 1.5 hours after thawing cells were stained by TMRM as described in *Materials and Methods*. Mean FI and FI SD values were measured by Image Analysis in each individual cell. The average mean and SD for each measured parameter were calculated for the different cell populations under investigation (controls and experiments) as described in *Statistical analysis.*Values are average ± SD of each group of cryo-devices.

### Cytoplasm membrane integrity and metabolic activity of U937 cells after the freezing - thawing cycle

**Figure 5:**
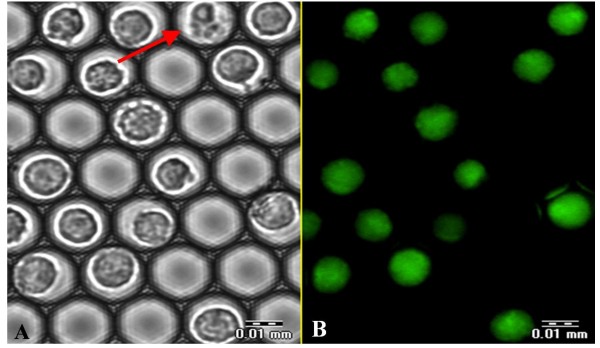
**Monitoring U937 cell membrane integrity after the freezing - thawing cycle by FDA staining**. The U937 cells were exposed to the freezing - thawing cycle in the i3C. At 3 hours after thawing, FDA solution (1.2 μM) was introduced into the i3C and images were taken 5 minutes after FDA addition. The transmitted image (A) and corresponding fluorescent image (B) are presented. The dead U937 cell unstained by FDA is indicated by the red arrow. Scale bar: 0.01 mm.

The intensity and rate of accumulation of the fluorescent product of FDA hydrolysis within the cells reflect both the enzymatic activity required for the hydrolysis and the integrity of the cell membrane necessary for the retention of the product. As a rule, the FDA staining is used for post-thawing estimation of cell vitality, since in the cells with damaged membranes, the accumulation of hydrolyzed fluorescein does not take place and the intracellular fluorescent signal is not observed [21,22]. Figure 5 presents a representative FDA staining image of U937 cells exposed to the freezing - thawing cycle in the i3C, at 3 hours after thawing. The figure demonstrates good intracellular accumulation of fluorescein in the majority of cells and the presence of only one non-stained cell. Moreover, it is clear that this membrane-compromised cell can be easily distinguished from the remaining, normal stained cells, based on its morphological appearance in the transmitted light image.

**Figure 6:**
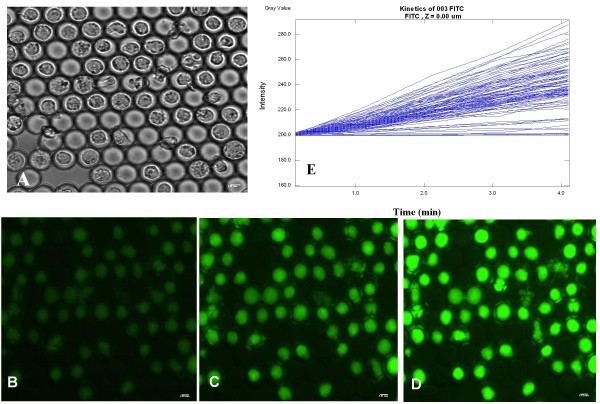
**Metabolic characteristics of U937 cells that underwent a freezing and thawing cycle in the i3C**. At 3 hours after thawing the cells were stained by FDA (1.2 μM) and the kinetics of FI increase were measured. Transmitted light image (A) and the corresponding fluorescence images (B-D) of U937 cells at 1 minute (B), 3 minutes (C) and 5 minutes (D) after FDA addition. Scale bar: 0.01 mm. (E) Intracellular staining rates derived from image analysis of *the same *U937 cells as in A-D. Each line represents the FI vs. time for an individual cell.

**Figure 7:**
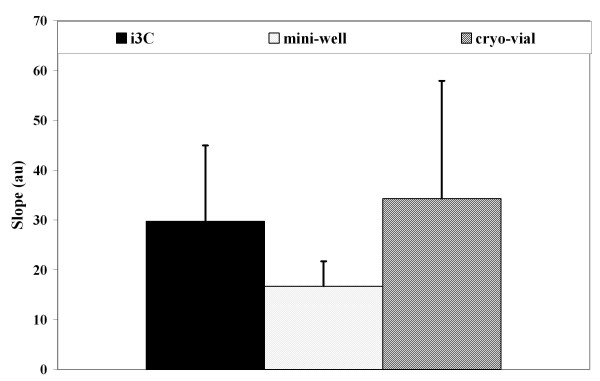
**Fluorescein diacetate hydrolysis rates at 19 hours after thawing**. For the evaluation of the metabolic recovery after the freezing - thawing cycle, the cells were stained by FDA and the linear slope FI(t) was calculated for each single cell by image analysis. The mean slopes for U937 cell groups that underwent the freezing - thawing cycle in the cryo-devices (at least 4 experiments for each cryo-device) are presented as mean ± SD.

Unlike PI and Annexin V staining, FDA staining of post-thawed U937 cells, as reflected only by the percentage of FDA positive cells, did not change significantly during the post-thawing incubation. The percentage of stained cells was 97.2% ± 2.8 and 89.8% ± 11.1 after 3 and 24 hours post-thawing in the i3C, and 89.3% ± 10.3 and 97.5% ± 3.5 for the corresponding time periods for cells frozen in control cryo-vials. This indicates that this parameter is not a sensitive marker for post-thawing cell status. The quantitative analysis of the individual cell rate of FDA hydrolysis was done by kinetic measurements of fluorescein accumulation and the calculation of the linear slope of FI increase over time [23,24]. Figure 6(A-D) shows the FDA staining of U937 cells at 3 hours after thawing, and the calculated staining rates (Figure 6E). As seen, there is a large difference in the linear slopes for individual cells within the entire cell population, which signifies a large variation in the individual metabolic activity. The percentage of post-thawed cells that exhibited a positive FDA hydrolysis slope was 77.1 ± 7.8 as compared to 86 ± 3.5 in the unfrozen cell population. The mean linear slope for the FDA staining was 60.2 ± 59.4 in the i3C-frozen cells, very close to that of the unfrozen U937 cells (62.4 ± 24.5). Figure 7 shows the mean FDA hydrolysis rates in the i3C-frozen U937 cells and in cells exposed to the freezing - thawing cycle in the control cryo-devices, as measured 24 hours after thawing. The statistical differences of the FDA hydrolysis rates in the cells that underwent the freezing - thawing cycle in the different cryo-devices were estimated at *P =*0.5.

### Multi-parametric analysis following the freezing - thawing cycle in the i3C

The unique optical and mechanical features of the i3C enabled the observation and treatment of the same individual cells, and finding direct associations between several parameters at a single cell resolution.

**Figure 8:**
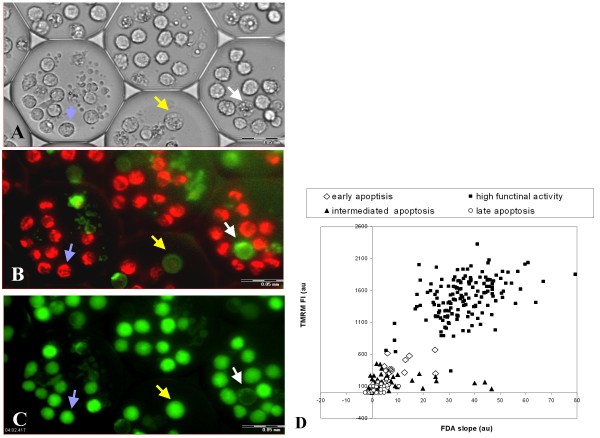
**Multi-parametric staining of cryopreserved U937 cells**. U937 cells were loaded into 100 μm i3C and underwent a freezing - thawing cycle. After 24 hours of post-thawing culture, the U937 cells were stained sequentially first by TMRM and Annexin V and next by FDA and PI. Transmitted light (A) and fluorescent images of U937 cells stained with TMRM and Annexin V (B), and FDA (C) are presented. Arrows indicate different cell statuses: Blue - high functional activity - morphologically intact, highly TMRM and FDA positive, Annexin V negative; Yellow - early apoptosis - a morphologically changed cell with a slightly condensed nucleus, exhibiting low TMRM staining, FDA positive staining and strong Annexin V staining; White - late apoptosis - a morphologically changed cell with nucleus defragmentation and pronounced Annexin V positive staining, TMRM and FDA negative staining. Scale bar: 0.05 mm. (D) Scatter diagram of individual TMRM (FI) values vs. esterase enzymatic activity values (slopes), measured in post-thawed U937 cells 24 hours after cell thawing. Note the detection of 4 cell subgroups based on their functional activity parameters.

U937 cells underwent the freezing - thawing cycle within 100 μm diameter i3C devices and then parallel analyses of mitochondrial membrane potential, intracellular esterase activity, and apoptosis levels at the resolution of individual cells, were accomplished using multi-parametric staining by TMRM, FDA hydrolysis rates, and Annexin V - PI test, respectively, as described in *Materials and Methods*, 24 hours after thawing (Figure 8A-C).

According to the established single parameter apoptosis assessment using double Annexin V and PI staining, three cell subgroups were identified:

**a. **The intact cell population (live cells) - negative for both Annexin V and PI staining.

**b. **The late apoptosis cell group as evidenced by the parallel staining with both PI and Annexin V.

**c. **Early apoptosis cells characterized by Annexin V binding but which were PI negative.

Utilizing the i3C methodology, additional analyses of MMP and esterase enzymatic activity in the same individual post-thawing U937 cells from the above cell groups were made. The percentages of the different cell groups and their corresponding average fluorescence signals are summarized in Table 4, while single cell results are illustrated in Figure 8D, in a scatter diagram of the individual cells' values of TMRM fluorescence intensity vs. esterase enzymatic activity.

**Table 4:** U937 cells' functional status after the freezing - thawing cycle.

Parameters measured (au)		Cell status and subgroups			
	
	Live cells (62%)	Late apoptosis (18%)	Early apoptotic cells		
			
			Total (19%)	MMP (+) (5%)	MMP (-) (14%)
**TMRM mean FI**	1476 ± 331	Negative	242.7 ± 155.6	429 ± 131	Negative

**TMRM FI SD**	652 ± 249	Negative	97.2 ± 11.2	141 ± 47	Negative

**FDA slop**	36.1 ± 11.36	Negative	9.6 ± 11.2	11 ± 7.1	9.05 ± 12.2

U937 cells underwent a freezing - thawing cycle as described in *Materials and Methods. *Overnight after thawing the multi-parametric analysis was performed and four parameters were elicited for each single cell. The cell subgroups were defined as described in *Multi-parametric analysis following the freezing - thawing cycle in the i3C *section. Values are average ± SD in individual cells' subgroups. The percentage of each subgroup from the total population in a representative experiment is shown in brackets.

As Table 4 shows, the late apoptosis cell group (17% of the post-thawing cells) showed no TMRM staining and no esterase activity. The intact cell group, which comprises the majority (62%) of the cell population, is characterized by high functional activity as reflected by the maximal TMRM FI and the highest FDA slopes. As shown in Fig 8D, within this cell cluster, the most vigorous cells are exemplified by the uppermost mitochondrial and enzymatic activity and can be easily selected.

Post-thawing cells that underwent early apoptosis (characterized by Annexin V binding, but which were negative for PI), display low mitochondrial and enzymatic activity. However, in this cell group, two cell subsets were detected based on the quantitative measurements of individual MMP values. The majority of this cell group (15% of the total cell population) showed no mitochondrial activity (TMRM negative) and low FDA hydrolysis rates and termed MMP ^- ^subgroup. An additional subset (5% of the total cell population) exhibited detectable but low mitochondrial membrane potential (< 30% of the mean FI for the cell population) along with low FDA hydrolysis rates (< 30% of the mean slope for the cell population). This subgroup termed early apoptosis MMP ^+ ^subgroup, and could be distinguished from the other apoptotic cells by their TMRM FI values (Figure 8D).

In untreated U937 cells growing in culture, the percentage of cells undergoing spontaneous early and late apoptosis was 12% ± 4% and 5% ± 3% respectively. Among the early apoptotic subgroup, most of the cells (9% of the total cell population) were MMP^+ ^and showed considerable mitochondrial activity.

## Discussion

In recent years, great effort has been dedicated to improving procedures for freezing and resuscitation of various cell types and to overcoming or minimizing the cell damage induced by freezing and thawing [25-27]. Each new approach calls not only for the optimal technical conditions, but also for noninvasive, simple, and reliable means to test the outcome of the process. The current work presents the means and methodologies for using an optical cryo-apparatus for monitoring several fundamental functional activities of cryopreserved cells, utilizing the recently developed i3C cryo-device.

Our results confirmed that spatially identifiable cells that undergo a freezing - thawing cycle in i3C devices exhibit the same functional activity as control cells. The proportions of intact cells and apoptosis rates, as well as the division of cells frozen and thawed in the i3C, were similar to those for cells that were frozen under the same experimental conditions in standard cryo-vessels. Moreover, the cells displayed a normal functional activity, as reflected by the multi-parametric quantitative analysis of the mitochondria status and intracellular enzymatic activity.

The use of cryopreserved cells is becoming widespread in drug screening, since cryopreservation *per se *has been shown to have minimal effects on pharmacological testing and can be applied to many cell types and cell assays, including arrested cell division [28]. However, in the fields of reproductive medicine and cell-based therapy, the ability of the post-thawing cells to divide and proliferate is crucial. The large picowells (100 μm) of the i3C enable us to culture and visualize multiple small cell groups, as well as monitor their behavior and propagation at a single cell resolution. With only a few cells in each picowell, real-time, kinetic measurements of cell division can be performed, thus specific non-dividing or highly proliferative cell clusters can be detected. This attribute is essential for stem cell clone analyses, where, after cell divisions, the newly formed compact colony remains confined to its picowell, enabling time-lapse imaging of the evolving clone. Moreover, the unique optical features of the i3C facilitate the morphological observations of cryopreserved cells in bright field microscopy. Such a non-intrusive, label-free technique is imperative for the future utilization of the post-thawed cells in clinical applications.

It has been shown that low mitochondrial activity is associated with cryo-damage [15]. Mitochondrial dysfunction in porcine oocytes was found to be directly responsible for the early arrest of pre-implantation embryos *in vitro *[29]. An irreversible loss of high mitochondrial membrane potential was detected in cryopreserved mouse embryos [30]. The decrease in MMP was found to be involved in the activation of caspase-3 and apoptosis induction [31].

In our cell system, the post-thawing appearance of TMRM-negative cells probably reflects the total collapse of MMP, which was seen upon opening the mitochondrial permeability transition pore during apoptotic signaling [19]. However, time dependent recovery of the mitochondrial trans-membrane potential as manifested by the increase in TMRM staining of post-thawed cells 24 hours after FTC, was shown in the rest of the cell population. These results are in agreement with previous data [32].

Furthermore, within TMRM-positive cells, the assessment of individual cells' MMP by temporal and spatial quantitative analysis of the fluorescence signal was found to be a very sensitive parameter for cell status following the freezing - thawing cycle. Maximal TMRM signals accompanied fully functioning cells that were Annexin V and PI negative and showed the highest FDA hydrolysis rates. Multi-parametric analysis at the individual cell resolution is the most significant achievement of the methodology presented here. Simultaneous quantitative measurement of single cells' MMP, esterase activity, and phosphatidylserine externalization, was used to detect the most viable and active cells within the cryopreserved cell population. All of the above parameters are well-established apoptosis markers.

The role of apoptosis in the low recovery rates after cryopreservation of human embryonic stem cells [10] and of hematopoietic stem cells and progenitor cells [33] has recently been emphasized. As a consequence, new strategies to enhance the post-thawing survival of such precious cells, by utilizing either protease or apoptosis inhibitors have emerged [33,34]. Since different cell types, at different cell development stages, have varying sensitivities to cryopreservation-induced apoptosis, the ability to assess the rate of apoptosis in individual post-thawed cells is crucial.

Moreover, the variability of the kinetics of the death course within cell populations calls for monitoring several apoptotic parameters occurring in single or subgroups of cells within a complex population.

The presented i3C device and methodologies address the above requirements for multi-parametric evaluation of the program cell-death process at the level of individual cells. The system enables not only the detection of fully active and apoptotic cells by the traditional Annexin V - PI staining, but the depiction of individual cells and subgroups by the additional simultaneous analysis of MMP and esterase activity. Thus, the quantitative measurements of individual cells' TMRM FI and FDA hydrolysis rates revealed apoptotic subpopulations which would have been undetected by other methods. Moreover, among the heterogeneous population of viable cells, which exhibited variable metabolic and mitochondrial activities, the selection of the most promising active cells becomes feasible.

## Conclusions

In conclusion, we introduce advanced technological and analytical capabilities for fluorescence and bright-field investigation of individual post-thawed cells. Such an examination reveals the functional and morphological features of each cell within the population and facilitates the detection and subsequent retrieval of the most viable, highly active cells for further utilization.

## Competing interests

The authors declare that they have no competing interests.

## Authors' contributions

EA led this study and its experimental design, carried out fluorescence measurements during the FTC, participated in the process of image analysis, and drafted the manuscript together with MD. MD conceived the individual cell based Cytometry approach, supervised this study and, together with EA, drafted the manuscript. MD, together with HZ, designed and coordinated this study. AD, together with SH, contributed to the microstructure design and manufacturing aspects of the i3C. FE participated in the experimental design, carried out fluorescence measurements during the FTC, and performed the literature survey. MG participated in performing the complementary control (routine) FTC experiments. IM was responsible for the various regimens of FTCs and relevant controlling S/W programs, and contributed to the analysis of images. SM prepared the i3C devices and tested their performance during FTC. YN programmed the S/W which automatically identified populated/empty picowells, stained and unstained cells in the picowells, and prepared the acquired data for the 2^nd^/3^rd ^party analysis. YS participated in the experimental design, carried out fluorescence measurements during the FTC, and participated in the image analysis. MS carried out fluorescence measurements during the FTC and participated in the image analysis. MT, together with HZ, identified the advantages of the individual-cell-based cryopreservation approach for cryo-medicine. HZ conceived the individual-cell-based cryopreservation approach and, together with MT, identified its advantages for cryo-medicine. NZ supervised the biological and image analysis parts of the study, participated in designing the study and drafting the manuscript. All authors read and approved the final manuscript.
